# Stem cell therapy: light in the tunnel for penetrating Crohn's disease

**DOI:** 10.1093/gastro/goac085

**Published:** 2023-03-25

**Authors:** Nan Lan, Xianrui Wu, Bo Shen

**Affiliations:** Center for Inflammatory Bowel Disease, Columbia University Irving Medical Center/NewYork Presbyterian Hospital, New York, NY, USA; Department of Colorectal Surgery, The 6th Hospital of Sun Yat-Sen University, Guangzhou, Guangdong, P. R. China; Center for Inflammatory Bowel Disease, Columbia University Irving Medical Center/NewYork Presbyterian Hospital, New York, NY, USA

**Keywords:** Crohn's disease, fistula, perianal disease, stem cells, therapy

## Abstract

Patients with Crohn's disease frequently suffer from fistula resulting from adverse sequelae of persistent complicated active disease or surgical intervention. Fistula affects a patient's quality of life and is directly associated with the need for surgical intervention. Diagnosis of fistula can be made through CT enterography, MR enterography, gastrograffin-based imaging, and transanal ultrasound. Treatment for fistula mainly consists of medication, endoscopic procedures, and surgery. There are emerging approaches under current investigation, such as stem cell therapy. The results showed a decent response in patients with perianal and rectovaginal fistula with minimal side effects. Further investigation is still needed for other internal fistula.

## Introduction

Crohn's disease (CD) is a chronic inflammatory and disabling disease. Patients with CD frequently suffer from fistula/abscesses that can result from adverse sequelae of persistent active mucosal and transmural inflammation or surgical intervention [1]. The reported frequency of spontaneous fistulas associated with active disease, including the perianal fistula, enteroenteric fistula (EEF), enterocutaneous fistula (ECF), rectovaginal (RVF) fistula, and enterovesical (EVF) fistula, ranged from 14% to 50% of CD patients [2–4]. A fistula that often leads to abscess adversely impacts a patient's quality of life and results in surgical intervention.

The pathophysiology of fistula formation is still poorly understood. One theory was an epithelial-to-mesenchymal transition (EMT), which refers to the mechanism by which epithelial cells lose their essential epithelial-defining properties while gaining the qualities of mesenchymal cells. EMT promotes epithelial integrity and re-establishes the mucosal barrier, which might be an attempt of the intestinal non-immune cells to close the mucosal defects and might play an important role in the formation of CD fistula. The penetrating disease was previously proposed also to be related to ulcers or transmural fissures that gradually penetrate the surrounding soft tissue and eventually communicate with the other bowel segments or organs such as the bladder, vagina, and skin [3, 5]. Immune-mediated pathogenesis of CD and molecular genetics are also related to the primary formation of fistulas [6]. Therefore, understanding the possible underlying etiology and pathogenesis would be beneficial in developing treatment options. Reported treatment options include medical management, endoscopic management, and surgical management. Medical treatment is often limited due to the structural nature of the fistula and abscess. Antitumor necrosis factors (TNFs) can be effective if there is concurrent inflammation [6, 7]. Surgical treatment has previously been the standard of treatment; however, the disadvantages of surgery are invasiveness, inadequate efficacy, post-operative complications, and post-operative recurrence. Endoscopic treatment using modalities such as fistulotomy, incision, and drainage has evolved to be a valid option and has been explored [8].

On the other hand, stem cell therapy has been a developing interest in the treatment of CD-related fistulas. Many trials have revealed promising results using topical stromal cell and stem cell therapy. This review aims to discuss the recent development in the field of stem cell therapy in the treatment of CD-related fistulas.

## Diagnosis

The diagnosis of a fistula depends on the combination of clinical, radiographic, and endoscopic presentation. Symptoms for a CD-related fistula are dependent on the location of the fistula. While patients with EEF may be asymptomatic, patients with ECF likely have drainage from the fistula site. A passage of stool and gas through the vagina can be seen in RVF. Recurrent urinary tract infection and pneumaturia can be seen in EVF. In a fistula leading to an abscess, the patient may present with sepsis. Radiological evaluation is the recommended method for the characterization of fistulizing disease and other intra-abdominal penetrating diseases. Available radiological evaluation includes cross-sectional imaging such as fistulography, computed tomography (CT), and magnetic resonance imaging (MRI) [9, 10]. For a perianal fistula, examination under anesthesia (EUA) and transrectal ultrasonography help to diagnose and manage [11, 12].

### Cross-sectional imaging

Cross-sectional imaging techniques using enterography protocols with either CT enterography (CTE) or MRI enterography (MRE) and small intestine ultrasonography can be used to identify and quantify transmural structural damage and disease activity [13, 14].

Conventional CT of the abdomen with oral and intravenous contrast occasionally may obscure pathological mural enhancement [15]. CTE is tailored to maximize small bowel wall assessment. The oral neutral enteric contrast has the attenuation of water and thus increases the conspicuity of the enhancement of actively inflamed bowel wall following administration of intravenous contrast material [16, 17]. CTE has high sensitivity and specificity in detecting small bowel disease in CD [18, 19]. The diagnostic accuracy is comparable with MRE, while CTE has a more consistent image quality in a shorter acquisition time [20, 21]. CTE is also used in the detection of penetrating disease and its high availability makes it decent first-line imaging in clinical practice to detect fistulas with an accuracy rate of >90% [21, 22]. The pool sensitivity and specificity were 70% and 97%, respectively, across five studies in the detection of fistulas [23]. The disadvantage would be the large dose of x-rays that patients must undergo during each evaluation.

MRI, specifically MRE, has been widely utilized in the diagnosis of CD activity and complications such as strictures, fistulas, and abscesses. The process also requires the patient to ingest oral contrast in combination with intravenous contrast. In addition, spasmolytics help reduce bowel peristalses to minimize motion artifacts. Because of the lack of ionizing radiation, many sequential acquisitions can be obtained before and after injection, followed by dynamic imaging. MRE has similar sensitivity and specificity compared to CTE in the detection of fistulas. Pooled sensitivity and specificity were 76% and 97%, respectively [23]. MRE also has superior soft-tissue contrast resolution enabling a superior visualization of the inflammatory and fibrotic characteristics of the bowel wall. The disadvantage of MRE is the lack of standardized protocol. Scoring systems such as the Magnetic Resonance Index of Activity (MRIA) [24, 25] and Lemann score [26] have been used and validated to assess the treatment efficacy in CD patients but may not be as helpful in patients with penetrating disease.

### Gastrografin imaging

Gastrografin is a water-soluble, high-osmolality contrast medium that has been used diagnostically and occasionally therapeutically in obstructive disease. The use of gastrografin has been reported but not extensively studied in CD-related fistulas. Gastrografin enema has been utilized in the pouch patient. The sensitivity and specificity of detecting fistula are 33% vs 96% [28]. The accuracy of the combination of gastrografin enema and MRI was 71.4% and the combination of gastrografin enema and pouchoscopy was 100% [27]. Similar, gastrografin enema could potentially be a reliable diagnostic tool for perianal fistulas in patients with CD. One small study reported preoperative use of gastrografin via the anus in patients with chronic fistulas. The study included 27 patients, and 13/27 patients had fistulograms that revealed unexpected pathology or directly altered surgical management [28]. To enhance imaging, gastrografin has been added to modified CTE to evaluate small bowel obstructions. Its use has not been verified in the case of fistulas, leaks, or abscesses. In one study, gastrografin enema was used to diagnose EVF and no EVF was diagnosed; however, EEF was seen in two cases. Gastrografin enema is also often used prior to ileostomy reversal to determine the integrity of the anastomotic site [29].

Patient factors may make one imaging modality a better choice than another. For example, in patients with renal insufficiency, we would avoid CTE or MRI and instead favor other diagnostic modalities such as gastrografin enema due to the effect of contrast and gadolinium on the kidney. Future studies are needed to assess the efficacy and accuracy of gastrografin enema in CD-related fistulas.

### Ultrasonography

Conventional transabdominal ultrasound is effective in visualizing the bowel wall layers. Therefore, bowel thickness, mural stratification, luminal narrowing/stenosis, and/or bowel dilation can all be observed. The sensitivity of detecting CD is high in the experienced centers. However, no reports specify the sensitivity and specificity of internal fistula detection. The use of Doppler sonography provided more information regarding the blood flow of the bowel wall, thus making ultrasonography a valuable tool to assess response to medication in CD patients.

A contrast-enhanced ultrasonography protocol includes the administration of intravenous contrast. The contrast agents demonstrate tissue perfusion with time blood-pool imagining. It was shown to perform better than gray-scale ultrasonography or color Doppler at CD detection. It has been more extensively studied and used in the European community. The use of intra-cavitary contrast-enhanced ultrasonography has a reported sensitivity and specificity of 87% and 100%, respectively [30].

Small intestine contrast ultrasonography is another modification of unenhanced gray-scale ultrasonography involving the ingestion of oral contrast. This has been shown to improve the detection of proximal small bowel disease and strictures. The sensitivity and specificity for the detection of intra-abdominal fistulas were 78.5%–96.0% and 66.7%–95.5%, respectively [31–33].

Transrectal ultrasonography is used in the assessment of CD-related perianal disease. It is performed with or without the administration of bubble-producing hydrogen peroxide. The reported diagnostic accuracy of this approach ranged from 56% to 90% [34]. There seems to be excellent consistency between MRI and transrectal ultrasonography in the diagnosis of perianal fistulas, with the latter being superior for the classification of transsphincteric and rectovaginal/anovulvar fistulas in experienced hands [35, 36].

Ultrasonography has the advantage of portability, availability, and improved tolerance. However, this procedure is highly operator-driven with variable quality and consistency.

### EUA

EUA has been considered the most sensitive diagnostic modality for perianal disease, with a reported accuracy of 90% [3, 37]. EUA is toned to be performed by experienced colorectal surgeons and allows the delivery of concomitant therapy such as seton or drainage placement, plug placement, localized injection, and stem cell therapy [38]. The role of EUA and other radiographic evaluations such as MRI is complementary [39]. This is because MRI and transrectal ultrasonography can provide more details regarding the fistula and surrounding structures, while EUA will be able to provide treatment [40]. Therefore, if an abscess is suspected of requiring drainage, EUA is recommended and should not be delayed, reducing the risk for branching fistula and further septic presentation.

## Classification of Crohn’s fistulas

A fistula is defined as a pathological connection adjoining two epithelialized surfaces [41]. For example, they can connect a portion of the intestine to the outer surface or another inner surface of another hollow organ. The classification of a CD-associated fistula is proposed in Table 1 [42]. There is no consensus on the classification of a CD fistula, but it is often first classified according to the location of the fistula in a perianal fistula, ECF, EEF, RVF, or EVF.

**Table 1.** Classification of inflammatory bowel disease-associated fistula [42]

Category	Subcategory	Example
Etiology	Primary or disease-associated	Crohn's disease-associated enterocutaneous fistula
	Secondary or anastomotic	Enterocutaneous fistula from ileocolonic anastomosis leak, parastomal enterocutaneous fistula
Underlying disease	Crohn's disease	Crohn's disease-associated jejuno-colonic fistula
	Ulcerative colitis	Mucus fistula from Hartmann pouch after subtotal colectomy
	Ileal pouch	Enterocutaneous fistula from the tip of the “J” of the pouch to the skin
Symptomatology	Dry	–
	Draining	–
	Abscess ±systemic symptoms	–
Organ involved	Gut-to-gut	Gastro–colonic fistula, ileosigmoid fistula, duodenocolonic fistula, pouch–pouch fistula
	Gut-adjacent hollow organs	Rectal–vaginal fistula, ileal pouch–bladder fistula, esophagobroncheal fistula
	Gut-to-skin	Enterocutaneous fistula
Length	Short	<3 cm
	Long	≥3 cm
Depth (from the lumen of fistula track to bowel lumen)	Shallow	<2 cm
	Deep	≥2 cm
Concurrent inflammation adjacent to the primary orifice of the fistula	Absent	–
	Present	–
Concurrent stricture	Absent	–
	Present	–
Complexity	Simple	Single track
	Complex	Multiple, branched, multi-exit, associated abscess
Malignant potential	Benign	–
	Malignant	Adenocarcinoma, squamous cell carcinoma

Perianal fistulas can be further classified according to the Parks classification, the St James's University Hospital classification, and the American Gastroenterological Association (AGA) classification (Table 2) [43–45]. The Parks classification describes five types of fistulas, which are distinguished according to surgical anatomy (superficial, intersphincteric, transsphincteric, suprasphincteric, and extrasphincteric) [46]. According to the AGA classification, perianal fistulas are divided into two categories: simple fistulas or complex fistulas [43]. A simple fistula is a superficial, intersphincteric, or transsphincteric fistula with a single external orifice and without complicating features (abscess, rectovaginal fistula, rectal or anal stricture), located below the dentate line. A complex fistula is an inter-, trans-, supra-, or extra-sphincteric fistula above the dentate line, which may have multiple external orifices or complicating features. Complex fistulas are encountered more commonly than simple fistulas in patients with CD.

**Table 2.** Classification of perianal fistulas [43–45]

Category	Definition
**Parks classification**	
Intersphincteric fistula	Lesions are confined to the intersphincteric space
Transsphincteric fistula	Leaves the intersphincteric space through the external anal sphincter
Suprasphincteric fistula	Passes through the intersphincteric space over the top of the puborectalis, tracks down the levator muscle before tracking the skin
Extrasphincteric fistula	Passes from the perineal skin and penetrates the levator muscle into the rectum
**AGA classification**	
Simple fistula	Low location Single external opening No evidence of perianal abscess, rectovaginal fistula, anorectal stricture
Complex fistula	High location Multiple external openings Concomitant perianal abscess, rectovaginal fistula, anorectal stricture, and active rectal disease or not
**MRI classification**	
Simple linear intersphincteric fistula	Lesions are confined to the sphincter complex Ischio-anal and ischiorectal fossae are clear
Intersphincteric fistula with abscess or secondary track	
Transsphincteric fistula	Any track or abscess within the ischiorectal fossa or levator plate
Transsphincteric fistula with abscess or secondary track within the ischiorectal fossa	
Supralevator and translevator disease	

**Table 3.** Local injection of mesenchymal stem cells (MSCs) in the treatment of Crohn’s disease-related fistulas

Study	Study design	Number of patients	Source of MSCs	Allo- vs autologous	Outcome (healed)
Garcia-Olmo 2005 [60]	Phase I, open-label, single-arm	Perianal/rectovaginal/enterocutaneous fistula: 5	Adipose tissue	Autologous	Complete: 75%
Garcia-Olmo 2009 [61]	Phase II, open-label, double-arm, randomized	Perianal fistula: 49	Adipose tissue	Autologous	Complete:
		MSCs + fibrin glue: 24			MSCs + fibrin glue: 71%
		Fibrin glue: 25			Fibrin glue: 16%
Portilla 2013 [67]	Phase I/IIa, open-label, single-arm	Complex perianal fistula: 24	Adipose tissue	Allogenic	24 weeks:
					Partial: 69%
					Complete: 56%
Cho 2013 [78]	Phase I, dose-finding trial	Perianal fistula: 10	Adipose tissue	Autologous	1 × 10^7^: 3/3 partial
		1 × 10^7^ MSCs: 3			2 × 10^7^: 2/3 complete
		2 × 10^7^ MSCs: 4			4 × 10^7^: 1/3 complete
		4 × 10^7^ MSCs: 3			
Panes 2016 [62]	Phase III, randomized, double-blind controlled trial	Complex perianal fistula: 212	Adipose tissue	Allogenic	24 weeks, complete:
		MSCs: 107			MSCs: 50%
		Placebo: 105			Placebo: 34%
					Complication:
					MSCs: 17%
					Placebo: 29%
Garcia-Arranz 2016 [75]	Phase I/IIa	Rectovaginal fistula: 11	Adipose tissue	Allogenic	52 weeks, complete: 60%
Barnhoorn 2020 [79]	Double-blind dose-finding study	Perianal fistula: 21	Bone marrow	Allogenic	Complete:
		1 × 10^7^ MSCs: 5			1 × 10^7^: 63%
		3 × 10^7^ MSCs: 5			3 × 10^7^: 100%
		9 × 10^7^ MSCs: 5			9 × 10^7^: 43%
		Placebo: 6			
Gutierrez 2021 [80]	Prospective nonrandomized phase I trial	Complex perianal fistula: 20	Adipose tissue	Allogenic	Complete: 69%
Garcia-Olmo 2022 [64]	Phase III, double-blind, randomized–controlled trial	Perianal fistula: 40	Adipose tissue	Allogenic	104 week, complete:
		Darvadstrocel: 25			Darvadstrocel: 56%
		Control: 15			Control: 40%

**Table 4.** Systemic stem cell for treatment of Crohn’s disease-related fistula

Study	Type of study	Number of patients	Source of MSCs	Allo- vs autologous	Outcome
Zhang 2018 [81]	Randomized–controlled trial	Stem cell: 41	Umbilical-cord MSC	Allogeneic	Significantly more decrease in CDAI, HBI, and corticosteroid dosage
		Control: 41			
Melmed 2015 [82]	Phase Ib/IIa study	Stem cell: 50	Human placenta-derived cells	Allogeneic	Clinical response: 36% (stem cell) vs 0% (placebo)
		Placebo: 16			
Dhere 2016 [83]	Phase I	Experimental: 12	Bone-marrow-derived MSC	Autologous	Clinical response: 5/11 (experimental)
		Control: 4			

MSCs, mesenchymal stem cells; CDAI, Crohn’s Disease Activity Index; HBI: Harvey–Bradshaw Index.

An ECF can result from underlying CD (primary), anastomotic leak, or ischemia (secondary). A disease-associated ECF responds better to medical therapy than an anastomotic leak-associated ECF [6]. An ECF can also occur in a patient with ileostomy or colostomy. The recognition of the internal opening of an ECF can be challenging, often requiring administration of betadine, methylene blue, or hydrogen peroxide, or probing with a soft-tip guide wire.

An EEF is classified according to two locations joined by the epithelialized fistula tract. The typical locations include ileocolonic, ileosigmoid, ileoileal, coloduodenal, and jejunoileal. Cologastric and coloduodenal are less commonly seen. The first name is often referred to as the origin of the fistula, which is where there is active disease. The second name indicates the anatomic location of the receiving organ. An EEF often co-exists with bowel inflammation around the primary orifice of the fistula and with stricture of the intestine distal to the primary fistula openings. The primary opening of the originating bowel can be insidious and difficult to identify. Therefore, cross-sectional imaging is often required. The secondary or exit orifice in the target organs typically has minimal or no inflammation surrounding the mucosa.

An RVF or anovaginal fistula is found in 5%–10% of female CD patients [46, 47]. It is essential to distinguish between an RVF and an anovaginal fistula as the treatment approach may differ. Very rarely, a fistula can also occur in the uterus and fallopian tubes. EVFs in CD are relatively rare and are primarily diagnosed by symptoms and imagining such as CT and cystography. A recto-urethral fistula may also occur, although rare and with a similar presentation to an EVF.

A fistula can also occur at the anastomotic site after surgery. The fistula is also named and classified according to the anatomic location of the origin to the feeding site. Apart from classification by location and organ involved, fistulas can also be classified by length, symptoms, concurrent inflammation, concurrent strictures, and malignant potential (Table 1).

We, the authors, also noticed an interesting association between strictures and fistulas. Often there is/are stricture(s) distal to the primary orifice of an ECF or EEF. For example, an ileosigmoid fistula usually co-exists with a stricture at the ileocecal valve or terminal ileum. In contrast, patients with severe perianal disease often have anorectal ring stricture proximately. Our clinical observation suggests that endoscopic treatment of the strictures helps to facilitate the therapeutic effects of concurrent medical therapy and maybe stem cell therapy.

## Rationale of stem cell therapy

There has been a growing interest in stem cell transplant in the form of mesenchymal stem cells (MSCs). Stem cells are undifferentiated cells that are auto-regenerating constantly. Stem cells can differentiate into different cell lineages depending on the local physical and biochemical conditions. MSCs can derive from bone marrow (BM-MSCs) or adipose tissue (AD-MSCs). MSCs participate in the regenerative tissue repair process and are activated by injuries or local inflammation. MSCs can promote local angiogenesis and proliferation of mesenchymal cells, and decrease the formation of useless scar tissue. In addition, they can modulate the local cellular and humoral inflammatory response, down-regulate T-cell activity, and increase IL-10 secretion [48]. As a result, they have an anti-inflammatory effect on the surrounding tissue [49]. All these characteristics of MSCs are being used to reproduce healthy copies of damaged tissues and replace them in the body of the patient. The anti-inflammatory effect on the surrounding tissue may be particularly important to healing CD-related fistulas. The immunosuppressive effect of MSCs in CD has been investigated in the *ex vivo* setting. It has also been reported that the MSCs from CD patients are no different compared to those of healthy patients in terms of phenotype, *in vivo* growth kinetics, and response to interferon γ. These findings provide an opportunity for autogenic therapy.

## Applications in perianal disease

### Systemic therapy

Locally administered MSCs were effective in multiple trials in CD patients with perianal fistulas. Intravenous MSCs were used in biologic-refractory luminal CD [50]. The study is a phase I open-label, single-arm trial that includes a total of 10 CD patients. Autologous bone marrow therapy was given through IV infusion and the outcome was evaluated using the Crohn’s Disease Activity Index (CDAI) and Crohn's Disease Endoscopic Index of Severity. None of the patients achieved complete remission, but 3/10 patients showed a clinical response [50]. A stage II open-label, double-arm randomized study was also conducted in 10 CD patients. Intravenous allogeneic MSCs led to clinical response at 14 days in three of nine patients with biologic-refractory CD [51]. Another stage II trial was conducted in Australia, where the allogenic BM-MSCs were given systemically with clinical remission in 8/15 patients at 42 days and improvement seen in 12/15 patients [52]. A few other studies also evaluated the use of systemic stem cell therapy showing similar results [53, 54]. A meta-analysis was conducted which showed that 40.5% of patients achieved remission after infusion of MSCs though there is high heterogeneity [55]. However, the data for systemic infusion in the treatment of CD-related fistulas have not been examined in detail. Most patients tolerated the procedure well, with only mild allergic reactions. One reported case of a dysplasia-associated lesion was found after MSCs treatment. However, the lesion may have developed prior to MSCs treatment; therefore, the causative connection cannot be established.

There are also reports on hemopoietic stem cell/bone marrow transplantation (HSCT) in CD patients [56, 57]. Autologous HSCT has been reported to benefit patients with severe refractory CD. However, due to its toxicity, it is not recommended in a majority of patients, especially given the development of biologics and small molecule agents [58].

### Topical therapy

**Figure 1.**
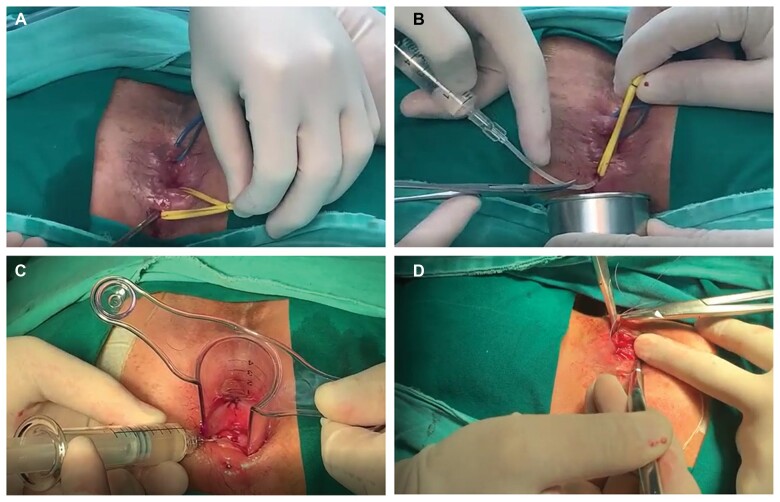
Topical stem cell therapy in a perianal fistula in Crohn’s disease. (A) Pre-existing seton and the fistula; (B) and (C) topical administration of stem cells; (D) mucosal flap to close the internal orifice of the fistula.

Stem cell therapy is currently performed in the operating room with patients under general anesthesia (Figure 1). The successful use of MSCs for the treatment of a refractory RVF in the setting of CD was first reported in 2003 by Garcia-Olmo *et al.* [59]. The same group conducted a phase I clinical trial in 2005, which included a total of five patients with CD-related fistula treated with autologous AD-MSCs injection. The results of the trial indicated that the protocol is feasible and safe, with a healing rate of 75% in both perianal fistulas/RVFs and ECFs [60]. The result soon generated a phase II randomized clinical trial within which patients were divided into two treatment groups receiving only fibrin glue and a combination of fibrin glue and autologous AD-MSCs. The reported healing rate was 71% in the combination treatment group and only 14% in the fibrin glue treatment group [61]. The double-blind phase III controlled trial, ADMIRE (Adipose-Derived Mesenchymal Stem Cells for Induction of Remission in Perianal Fistulizing Crohn's Disease), revealed a higher remission rate in patients receiving MSCs compared to the control group at Week 24 (50% vs 34%). Regarding safety, 66% of patients in the MSCs group and 65% in the control group experienced treatment-emergent adverse events, proctalgia, anal abscesses, and nasopharyngitis [62]. The long-term result of the same patients at 52 weeks showed a similar remission rate of 56% in patients treated with MSCs and 38% in patients treated with placebo, and complications such as anal abscesses and fistulas in 1 year were observed in 33% of the active and 29% of the placebo groups [63]. The most recent published data at 104 weeks continue to show a similar remission rate of 56% in the MSCs group and 40% in the control group [64].

Multiple other studies also dived into the same topic using autologous or allogenic, bone marrow, or adipose tissue MSCs [65–68]. There have also been variations between donors, making it difficult to generalize the findings of the studies. Immunophenotypes of BM-MSCs and AD-MSCs are 90% identical; therefore, the difference in efficacy is minimal. It seems that AD-MSCs have a higher and more prolonged replication rate in culture. AD-MSCs also have a greater anti-inflammatory and anti-angiogenic potential [69, 70]. AD-MSCs express even fewer HLA class-1 molecules and are also easier to access [70]. Between autologous vs allogeneic MSCs, autologous cells are usually preferred over allogenic for compatibility reasons. However, allogenic also has a role in the future as one batch can be used for a larger number of patients. It will also spare the patient from additional surgery, such as liposuction [71].

Meanwhile, fibrin glue was studied as a combination therapy with MSCs. There is insufficient evidence to recommend intralesional fibrin glue as a combination therapy. A meta-analysis showed that MSCs plus fibrin glue were more effective for fistula healing than fibrin glue alone (51% vs 29%; *P *=* *0.003) [72]. A modified technique for delivering MSCs with fistula plugs has also been investigated in the phase I study. The study included 12 patients who failed anti-TNF therapy and underwent intraoperative placement of the plug loaded with autologous MSCs. Complete fistula closure was achieved in 9 of 12 patients (75%) at 3 months and 10 of 12 patients (83.3%) at 6 months [73]. Another study combined AD-MSCs, platelet-rich plasma, and endorectal advancement flaps for the treatment of refractory perianal fistulas and showed a complete healing rate of 91% [74].

## Applications in other penetrating diseases

There has been much data on the treatment of perianal fistulas, but further investigation is still needed in intraluminal fistulas. Since the initial case report in 2003, there have only been three other studies in which the treatment of RVFs was discussed. The first was a phase I trial of nine patients treated with autologous AD-MSCs, of whom three had RVFs; two healed after injection of 30–300 million cells [60]. The second trial reported five patients with CD-associated RVFs who received allogeneic AD-MSCs; three of five patients achieved healing in 1 year [75]. The last study, which included five patients with RVFs treated with matrix-delivered autologous MSCs, reports improvement in all patients and three of five had complete resolution at 6 months [76].

ECFs have also been included in some studies, along with perianal fistulas. They have been individually studied in one trial by Garcia-Olmo *et al.* [77]. The study included four ECF fistulas treated with AD-MSCs and a cure was achieved in three-quarters of the fistulas. In a series that received stromal vascular fraction cells, healing of the fistula was found in only a quarter of cases.

**Figure 2.**
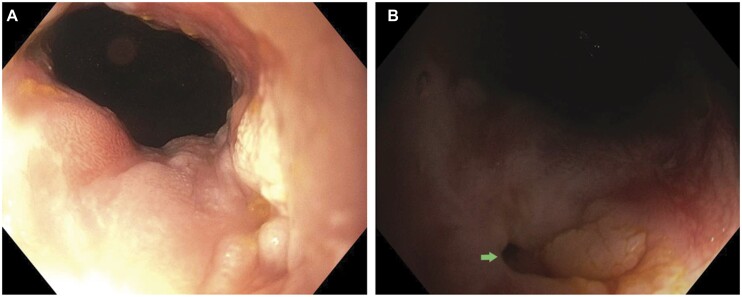
Recurrent pouch vaginal fistula after stem cell therapy. (A) Status of temporary closed pouch vaginal fistula at 3 months post stem cell therapy; (B) recurrent fistula (green arrow) 9 months after the therapy.

The use of MSCs in the treatment of EEFs, EVFs, and other penetrative diseases such as abscesses or sinuses has not been investigated. Further utilization of MSCs in other penetrating diseases warrants further investigation. MSCs have been used for the treatment of pouch vaginal fistulas, with dismal results so far (Figure 2).

## Summary and recommendations

A CD-related fistula is a common complication that significantly impacts the disease outcome and the patient's general quality of life. Fistulas in CD should be managed by a multidisciplinary team that includes a gastroenterologist, a colorectal surgeon, and a gastrointestinal radiologist. The diagnosis is mainly achieved through cross-sectional imaging using CTE or MRE. Treatment includes medications, endoscopic therapy, surgical therapy, and stem cell therapy. Stem cell therapy using MSCs has been a growing interest, with promising results in most clinical trials involving perianal fistulas. The more commonly investigated therapy has been localized treatment with allogenic AD-MSCs. In the future, we need trials to identify the ideal type of stem cell for different fistula locations and the dosage. In addition, investigations regarding the standard for outcome measures is essential, along with longer follow-up to evaluate the long-term outcome and perhaps the neoplastic complications from stem cell therapy.

## Authors’ Contributions

All the authors contributed substantially to discussions of the article content. B.S. conceived of the idea. N.L. drafted the manuscript. All authors read and approved the final manuscript.
